# Short-term exposure to some heavy metals carried with PM_10_ and cardiovascular system biomarkers during dust storm

**DOI:** 10.1038/s41598-023-31978-x

**Published:** 2023-04-15

**Authors:** Ahmad Badeenezhad, Iman Parseh, Ali Veisi, Saeid Rostami, Mousa Ghelichi-Ghojogh, Gholamreza Badfar, Fariba Abbasi

**Affiliations:** 1 Department of Environmental Health Engineering, School of Medical Sciences, Behbahan Faculty of Medical Sciences, Behbahan, Iran; 2 Department of Physiology, Behbahan Faculty of Medical Sciences, Behbahan, Iran; 3 Environmental Health Engineering, Shiraz University of Medical Sciences, Shiraz, Iran; 4 Department of Epidemiology, Golestan University of Medical Sciences, Gorgan, Iran; 5 Department of Pediatrics, Abuzar Children′s Hospital, Ahvaz Jundishapur University of Medical Sciences, Ahvaz, Iran

**Keywords:** Biomarkers, Cardiology

## Abstract

This study aimed to evaluate the effect of short-term exposure to heavy metals (HM) extracted from PM_10_ on CB in workers’ population in an outdoor space located in southern Iran during a dust storm. At first, 44 healthy and non-smoking workers were selected. Then PM_10_ and Blood samples were collected before and after the dust storm. Finally, HMs associated with PM_10_ measured by ICP-MS and its effect on the CB, including fibrinogen, CRP, TNF-α, and BP were estimated by ANOVA, Pearson correlation, and Odd Ratio (OR) in SPSS23. Based on the results, the concentration of PM_10_ and extracted HM such as Cr, As, and Cd was higher than the WHO/EPA standards in dust storms they increased the CB and BP remarkably. Moreover, the level of fibrinogen, blood pressure (BP) and TNF-α in dust storms were higher than in normal conditions (*p* < 0.05, OR > 3). In addition, As and Cd decreased fibrinogen concentration and systolic BP, respectively. Whereas, TNF-α was associated with concentration of Pb (R = − 0.85) on normal days. Consequently, the HM on PM_10_ such as As, interferes with the level of investigated CB. These results considered a potential risk for the residents in the southern regions of Iran.

## Introduction

Air pollution is an environmental and healthy challenge, particularly in developing countries. Some of the adverse effects and potential risks of air pollutants on human health are well-known. So, many epidemiological studies have clearly shown a significant relationship between exposure to air pollution and mortality rate^1–5^. Among the air pollutants, PM_10_ is considered one of the most important. Evidence is shown that these particles increased mortality rate, the incidence of respiratory and cardiovascular disease, etc. Moreover, PM_10_ reported that as an environmental risk factor for the COVID-19 outbreak^1,6^. In addition, these adverse effects can associate the age, gender, climate condition, and biochemical metabolism that are reported during both long-term and short-term exposures^7–13^. Particles also involve the respiratory tracts through direct and indirect paths^14,15^. Therefore, they penetrate the blood Circulatory system and then can damage other tissues^16,17^. The American Heart Association has argued that heart disease and mortality associated with PM may relate result of systemic inflammation and endothelial dysfunction^18,19^. Some studies have shown that exposure to PM_10_ has been significantly associated with cardiovascular and respiratory diseases in residents near open-pit mining regions^15,20–22^.

Besides, the adverse health effect of PM_10_ can associate with toxic substances such as heavy metals that adsorb on the surface of particles. So, they produce reactive radicals that damage the target tissue^8,23–25^. Some of these HMs can be very harmful at low concentrations, which may cause respiratory diseases such as bronchitis and asthma, carcinogenesis, or heart diseases^26–28^. Studies have shown that HMs destroy the protective antioxidant mechanisms that can classify as oxidative stress in human cells^11,29^.

These adverse effects also are observed after exposure to dust storms with a high concentration of PM_10_ that is occurred in semi-arid and desert regions such as the Middle East^10,30^. However, there is less attention to the effect of dust storms on workers with a specific physical activity. Most of them were focused on human health effects due to industrial particles^18,24^. Besides, there are no published studies to investigate the effects of HMs associated with airborne particles on the CB based on our best knowledge.

Therefore, we investigated the effect of short-term exposure to heavy metals carried with PM_10_ on CB and blood pressure in exposed workers to dust storms in the Middle East Dust Storm.

## Material and methods

### Study location

**Figure 1:**
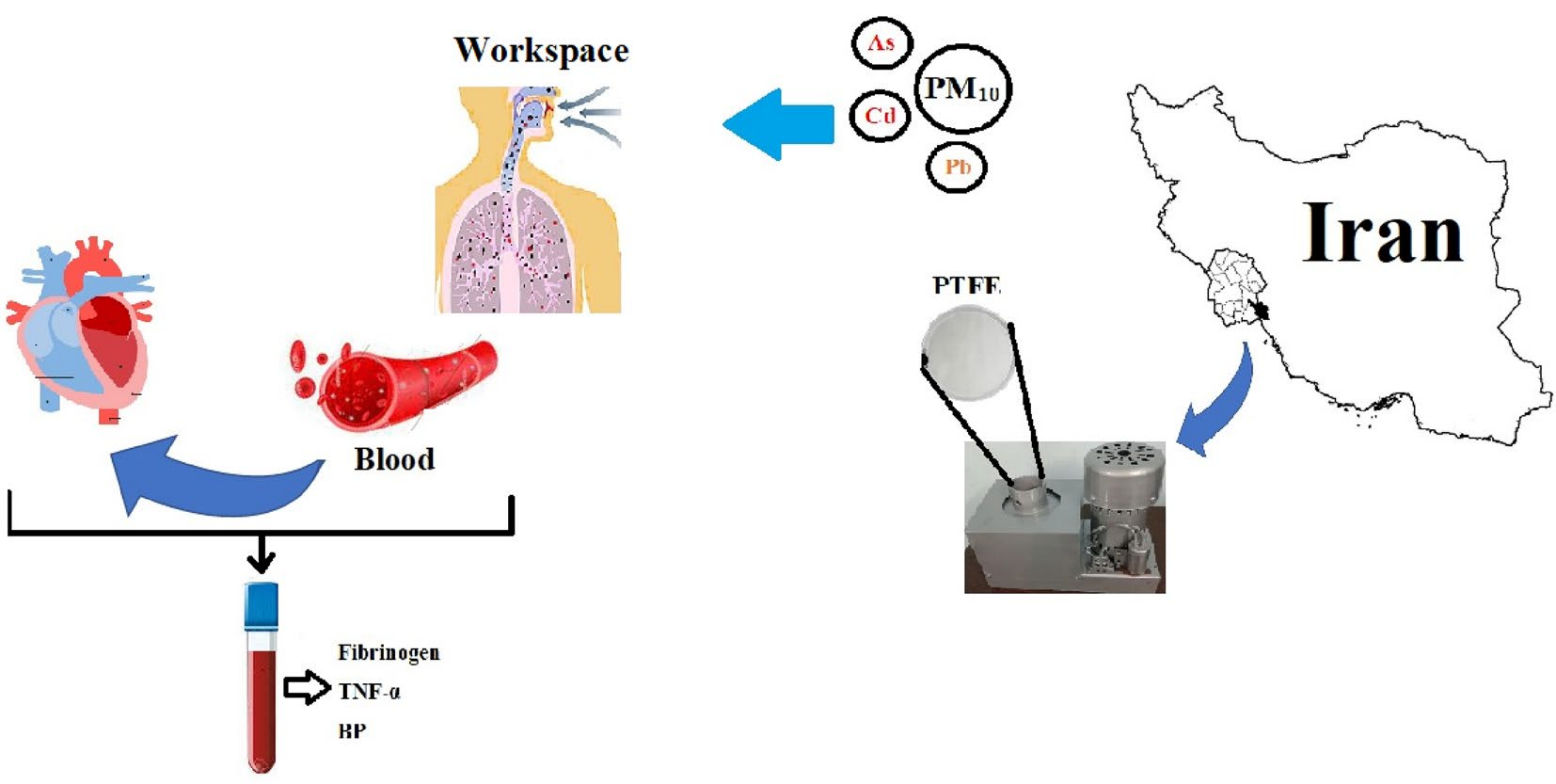
Schematic summarize of this Study and the its outcomes.

Behbahan city is geographically located in the southwestern region of Iran, with a hot and dry climate and relatively low rainfall. The blowing of dry and hot winds from the north and humid winds from the south in the summer resulted in unpleasant weather. In this city, the temperature is high, and the evaporation rate is considerable. The city’s height is 313 m above sea level, and its geographical coordinates are 50°14′ E and 30° 36′ N. In addition, Behbahan has relatively short distance from the deserts of the Middle East, which is the origin of many dust storms and can significantly affect the city. The schematic form of this study is presented in Fig. 1.

### PM_10_ sampling

In this study, airborne particle sampling was performed on normal and dusty storm days. Suspended particle sampling was carried out in an outdoor market located in Behbahan city center. Sampling was performed using a high-volume sampling pump (MEGA SISTEM LIFETEK 33 XP-R model). PM_10_ nozzle and polytetrafluoroethylene filters were used (47-mm ID, 0.5-mm pore size). The sampling duration was 24 h with 3-day intervals, and the input flow rate of 16 L/min in 2020 on both normal and dusty storm days. Filters were dried in a desiccator before and after sampling to remove moisture. Sampling was done at the height of 1.8 m from the ground and in the desired location without any obstacles reducing airflow amount. The filters were placed in a labeled plate with a specific code for each sample and were transferred to the laboratory in completely hygienic conditions.

### Determination of PM_10_ concentration

In this study, PTFE filters were weighed using a digital scale before and after sampling. Based on gravimetric techniques, the PM_10_ concentration was calculated through Eq. (1).

1 $$\mathbf{P}\mathbf{M}10=\frac{({{\varvec{W}}}_{{\varvec{f}}-}{{{\varvec{W}}}_{{\varvec{i}}})\boldsymbol{*}{10}^{6}}_{\boldsymbol{ }\boldsymbol{ }\boldsymbol{ }}}{{\varvec{V}}}$$

where PM_10_: PM_10_ concentration (µg/m^3^), Wf: filter weight before sampling (µg), Wi: filter weight after sampling, and V; the total sampling volume (m^3^).

For heavy metals concentration measurement (As, Cd, Cr, Cu, Fe, Pb, and Zn), each PTFE filter was split into tiny pieces, then transferred to a Teflon container. Then filters were digested by the three acids method. The hydrofluoric acid (1 ml), nitric acid (10 ml), and perchloric acid (HCLO_4_) (3 ml) were added to the Teflon container containing the crushed filter particles. Then, the suspension was heated in the oven at 170 °C for 4 h. In the next step, the cooled solution was dried at 95 °C, and 1 ml of nitric acid was added to the composition, and finally it was diluted with 10 ml of deionized water. Then, the concentration of heavy metals was measured using the ICP-MS 5100 with a detection limit of ± 0.01 ppb (Agilent Technology Company, Germany).

### Studied population

In the present study, some cardiovascular biomarkers variation was measured during short exposure to heavy metals on PM_10_. According to inclusion criteria, the cases were selected from young workers between 20 and 60 years. They were physically healthy humans. So, there were no diseases history such as cardiovascular, pulmonary, renal, liver diseases, or diabetes. Moreover, some properties of cases such as smoking, use of respiratory protective equipment, the dose of fast food, and exposure to other fume classified are expressed in Table S1. Then the exposure to dust storms was studied and monitored. The subjects were exposed to the dust storm for about 7 h that occupied in routine workplace, and none of them used protective equipment such as masks. All methods were performed by the relevant guidelines and regulations Ministry of Health Ethics Committee (IR.BHN.REC.1398.022).

Adherence to the scientific and research ethical standards according to the conditions of people's entry, sample collection, and follow-up of test results regarding incidents related to people's health were considered. The air sampling and biomarkers monitoring were performed on 44 participants before and after the dust storm. Blood samples were collected by certified staff from subjects 24 h before the dust storm between 8 and 10 am (PM_10_ < 100 μg/m^3^). The second sampling stage was done 24 h after the dust storm (PM_10_ > 100 μg/m^3^).

### Biomarkers detection

Cardiovascular biomarkers, including CRP, fibrinogen, and TNFα, were measured in the study. Before blood sampling, the systolic and diastolic blood pressure were measured and recorded along with other characteristics of the subjects. The questionnaire included confounding factors such as smoking status, exposure to toxic and industrial pollutants and the exposure duration, people's lifestyle including nutritional status, exercise, as well as physical and psychological health.

### Statistical analysis

Paired samples t-test analysis was used to analyze the changes in cardiovascular biomarkers and mean blood pressure before and after the test. Spearman correlation analysis was used to evaluate the relationships between variables. The difference between metals and biomarkers concentration before and after exposure was determined using the t-test. The Odd ratio was used to estimate the suffering from each of the examined outcomes chance. The confounding parameters effect from the questionnaire was determined by Correlate Partial test. The curves were drawn in Excel 2013, and analyzes were analyzed using SPSS 23.

### Ethics approval and consent to participate

This study was approved by the Medical Ethics Committee of the Ministry of Health and Medical Education (MOHME) of Iran (IR.BHN.REC.1398.022). Consent was given by all the Participates. Informed consent was obtained from the participants.

## Results and discussion

### The PM_10_ concentration

Table 1 is shown the concentration of PM_10_ during the normal and dust storm.

**Table 1:** The concentration of PM_10_ (μg/m^3^) before and after the dust storm.

Weather conditions	Average	SD	Max	Min
Normal days	89.25	12.33	118.4	63.7
Dusty days	291.5	25.44	344.6	275.55
Standard	100			

According to Table 1, the concentration of PM_10_ was 89.25 ± 12.33 and 291.5 ± 25.44 μg/m^3^, respectively in normal and dust storms that exceeded the WHO and EPA guidance. In the study of Heidari in Ahvaz, the PM_10_ concentration was 116 and 700 mg/m^3^ during the normal and dust storm, respectively ^3^. The PM_10_ concentration in this study was higher than in other regions of the world, including Thailand, China, America, and the European regions ^31^.

A possible reason for these differences might be related to the sharp increases in the condensed dust storm including PM_10_ in southern Iran and many regions of the Middle East. It caused the resident population experiences several dusty days a year in this geographical area. Thus, it is necessary to investigate the effects of these particles on people's health, especially in the cardiopulmonary system. In this study, the relationship between PM_10_ and several cardiovascular biomarkers has been discussed.

### The concentration of heavy metals on the PM_10_

**Figure 2:**
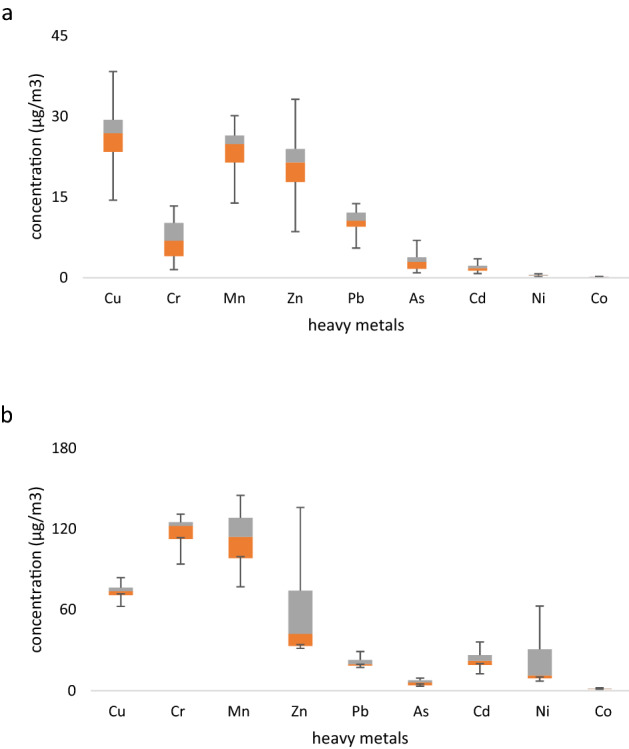
The concentration of heavy metals on PM_10_. (**a**) Normal day and (**b**) Dust storm.

One of the important challenges of PM is its high surface-to-volume ratio on other pollutants accumulation ^32^, such as heavy metals. Therefore, it is essential to evaluate the loading of heavy metals and their toxic effects. The concentration of accumulated heavy metals on the PM_10_ during the normal and dust storm are shown in Fig. 2.

According to Fig. 1, the average concentration of heavy metals including Cr, As, Cd, Pb, Cu, and Mg in normal day were 7.1, 3.19, 2.1, 10.5, 25.3, and 23.2 μg/m^3^, respectively. Also, on dusty days, the concentration of these metals was measured as 116.4, 113, 66.69, 65.66, 6.38, 23.58, and 21.79 μg/m^3^, respectively. Although the concentration of heavy metals was lower than the concentration in Ahvaz ^3^, the concentration of Cr, As, and Cd concentration on dust storm were higher than the EPA and WHO standards. Besides, the correlation between diastolic blood pressure and PM_10_ is higher than other factors (R = 0.52). In Los Angeles, exposure to PM_2.5_ during the 5-day increased the mean systolic and diastolic blood pressure approximately 3–13.4 and 3–6.8 mmHg ^2^.

### The profile of biomarker concentration and blood pressure

**Figure 3:**
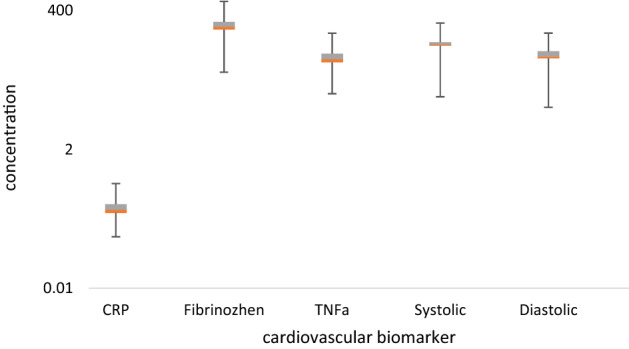
The distribution of cardiovascular factors in a normal day in the studied population.

**Figure 4:**
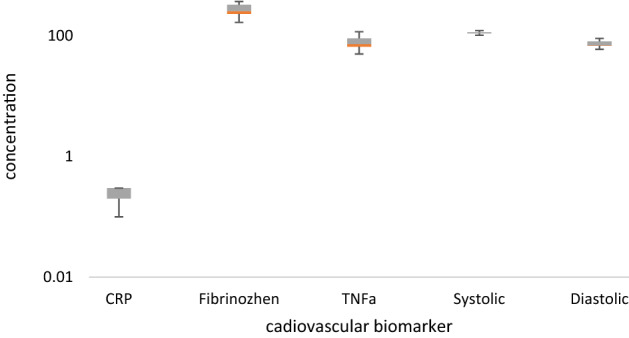
The distribution of cardiovascular factors in dust storm day in the studied population.

The distribution of the cardiovascular factors including CRP, fibrinogen, and TNF-α, as well as the systolic and diastolic blood pressure levels in normal and dust storm in the studied population are shown in Fig. 3 and 4.

According to Fig. 3, the mean concentration of CRP, fibrinogen, and TNF-α during normal day were 0.49 mg/l, 226.1 mg /dl, and 63.6 pg/ml, respectively. Also, the mean systolic and diastolic blood pressure before the exposure to dust storm were measured 110.8 and 70.3 mm Hg, respectively.

According to Fig. 4, the mean concentrations of CRP, fibrinogen, and TNF-α were 0.2 mg/L, 267.1 mg/dl, and 76 pg/ml, respectively, in the blood samples of population in dust storm day. The mean systolic and diastolic blood pressure during the exposure to dust storm were 112.4 and 72 mm Hg, respectively.

The results showed that the fibrinogen mean concentration (*p* = 0.014), the mean systolic (*p* = 0.01), and diastolic blood pressure (*p* < 0.0001) in the studied population in a dust storm were higher than on a normal day. The study by Liu et al. (2021) showed that the level of systolic and diastolic blood pressure in women exposed to heavy metals on the PM was higher than the normal conditions^9^.

Because, the epithelium of the respiratory tract modulated and promoted some inflammatory factors, including TNF-α due to exposure to airborne particles^14,23^. The results showed that the systolic and diastolic blood pressure of 11% of the total studied population was in the range of hypertension on a dust storm day. While it was estimated approximately 4% on a normal day. These results can be attributed to exposure to PM and its loading of heavy metals. Because the other risk factors and confounder variables such as nutrition, smoking, BMI, exposure to industrial pollutants, and duration of exposure to the toxic fumes were adjusted.

In addition, the odds ratios for increasing cardiovascular biomarkers, including fibrinogen, CRP, and TNF-α, as well as the mean systolic and diastolic blood pressure, are expressed in Table 2.

**Table 2:** Odds ratios for increasing of cardiovascular biomarkers and blood pressures.

Type of factors	Odd ratio	Lower	Higher
Systolic pressure	3.3	5.2	17.4
Diastolic pressure	2.1	1.41	12.1
Fibrinogen	0.19	0.31	0.75
TNF-α	3.1	2.3	31.5
CRP			

According to Table 2, the OR of fibrinogen (OR = 0.19, 95% CI 0.31, 0.75), TNF-α (OR = 3.1, 95% CI 2.3, 31.5), and also mean systolic (OR = 3.3, 95% CI 5.2, 17.4), and diastolic (OR = 2.1, 95% CI 1.41, 12.1) blood pressures were increased due to exposure to PM_10_ in the dust storm. It is apparent that the exposure to haze conditions has the highest effect on the mean systolic blood pressure and is associated with an increase of 1.6 mmHg (Table 2).

In the previous studies, coarse particulate matter caused an increase of 1.79 mmHg in systolic blood pressure (OR = 2.95) and 6.9 mm Hg in diastolic blood pressure. The odds ratio for hypertension due to long-term exposure to PM_10_ has been ignored (OR < 1)^24,25,33^. While another study in South Korea also showed that the increase in blood pressure in people exposed to pollutants with acute effects such as SO_2_ and CO was only 0.24 mmHg and 0.26 mmHg, respectively^26,34^. Although the effect of particulate pollutants is chronic, the odds ratio for increasing cardiovascular biomarkers is very high based on the results of the present study. Therefore, the cardiovascular risk can be significant during exposure to PM_10_ on a dust storm day in southern Iran.

### The correlations between heavy metals concentration in PM_10_

According to the coherence and the possibility of band formation between the elements, the correlation between the concentrations of heavy metals on the surface of PM_10_ was determined (Table 4).

According to Table 3, the maximum correlations between metals before exposure to PM_10_ are related to Cd and zinc (R = 0.96) and Cu and Mg (R = 0.88), because the standard electrode potential of Cu and Mg is 0.52 and − 1.05 Volts at the ambient temperature, respectively. In this condition, the band formation between them is possible that the fine surface of PM_10_ acts as a catalyst that reduces the activation energy ^35^. However, there was an inverse relation between Mg and Cd (R = − 0.75) because Mg substitutes for Cd during the photochemical reaction in the presence of the atmospheric suspended particles, and the increasing of Mg reduces the level of Cd ^28^. Furthermore, there is a significant relationship between the concentration of metals during the dust storm, especially for Cu and Mg (R = 0.95), Mg and As (R = 0.97), and Cu and As (R = 0.88). Also, a negative relationship was observed between the concentrations of metals during the normal day. Because the semi-metals form the N and P-type semiconductors in the silicon matrix that is very predominant in the dust storm emission in the Middle East region and therefore, it can vary their concentration ^36^.

**Table 3:** The correlations between heavy metals on the surface of PM_10_.

	Cu	Cr	Mn	Zn	Pb	As	Cd
After exposure							
Cu	1	–	–	–	–	–	–
Cr	− 0.08	1	–	–	–	–	–
Mn	0.81	0.05	1	–	–	–	–
Zn	− 0.27	− 0.26	− 0.67	1	–	–	–
Pb	0.12	− 0.128	0.39	− 0.26	1	−	−
As	− 0.05	− 0.32	− 0.124	0.19	− 0.66	1	−
Cd	− 0.37	− 0.31	− 0.75	0.96	− 0.22	0.074	1
Before exposure							
Cu	1	–	–	–	–	–	–
Cr	0.75	1	–	–	–	–	–
Mn	0.95	0.69	1	–	–	–	–
Zn	− 0.003	0.36	0.21	1	–	–	–
Pb	0.39	0.73	0.15	− 0.16	1	–	–
As	0.88	0.49	0.97	0.18	− 0.08	1	–
Cd	− 0.31	0.33	− 0.46	0.14	0.7	− 0.66	1

### The relationship between cardiovascular biomarkers and Heavy metals concentration

The correlation between cardiovascular biomarkers and the concentration of heavy metals on the PM_10_ during the exposure to normal day and dust storm are expressed in Table 4.

**Table 4:** The correlation between cardiovascular biomarkers and the concentration of heavy metals on PM_10_.

	CRP	Fibrinogen	TNF-α	Systolic pressure	Diastolic pressure
Before exposure					
Cu	0.57	− 0.21	− 0.48	0.27	0.019
Cr	− 0.07	− 0.17	0.34	− 0.64	− 0.4
Mn	0.26	− 0.02	− 0.57	− 0.11	− 0.15
Zn	0.001	− 0.06	0.35	0.33	0.17
Pb	0.02	0.34	− 0.85	− 0.22	0.27
As	− 0.34	− 0.4	0.37	0.3	− 0.12
Cd	0.033	0.085	0.34	0.37	0.2
After exposure					
Cu	− 0.025	− 0.45	0.56	− 0.14	**− **0.077
Cr	− 0.16	− 0.27	0.34	− 0.46	− 0.45
Mn	− 0.01	− 0.64	0.51	− 0.006	− 0.023
Zn	− 0.13	− 0.44	− 0.18	− 0.079	− 0.23
Pb	− 0.09	0.36	0.12	− 0.59	− 0.43
As	0.02	− 0.7	0.44	0.57	0.07
Cd	− 0.1	0.49	− 0.26	− 0.01	− 0.44

According to Table 4, there was only a negative and significant relationship between the concentration of TNF-α and lead (R = − 0.85). However*,* the highest correlation was estimated between the mean fibrinogen and the concentration of As (R = − 0.7), as well as the level of fibrinogen and Mg concentration (R = − 0.64). Because As produce the oxidant agents that can lysis the fibrinogen in the cell. Then it is increased the concentration of inflammatory factors and consequently increases the heart biomarkers^37,38^. Moreover, As increased the systolic blood pressure (R = 0.57). This finding is in accordance with previous studies^39,40^. Also, it directly interferes the risk factor of cardiovascular diseases such as vascular endothelium, smooth muscle cells, platelets, and macrophages^41,42^. Another component of PM is the concentration of Mg which is an essential cofactor for the activity of enzyme that regulate the normal human growth. However, its high concentrations interfere with the expression of up-regulating inflammatoryfactor^43^. Based on the results of present study, there was the positive relationship between the concentration of As and systolic blood pressure in normal days, but it inverted during the exposure to dust storm. On the other hand, the positive correlation between Cd and systolic pressure was higher than diastolic during the exposure to normal day; while during the exposure to dust, this relationship has become severely negative. In a study of Regenica et al. (2020), it was demonstrated that there was a relationship between Cd concentration and systolic blood pressure on normal days. However, a specific relationship has not been observed for diastolic blood pressure^44^. Moreover, the increasing of systolic and diastolic blood pressure was directly related to Cu^9^. Other effective factors can be related to the inverse relationship between Cd, and As that the high concentration of As effectively increases the diastolic blood pressure (See Table 4).

## Conclusion

This study was conducted to investigate the relationship between the levels of cardiovascular biomarkers and extracted heavy metals on the surface of PM_10_. The results of this study are shown that the concentration of PM_10_ during the dust storm is higher than the standard, and the concentration of Cr, As, and Cd are higher than the WHO and EPA standards. This high concentration of heavy metals varied the cardiac biomarkers. So, the systolic pressure of 11% of cases is classified in hypertension, and there was a significant relationship with diastolic pressure (R = 0.52). These changes have increased the OR of systolic blood pressure (OR = 3.3, 95% CI 5.2, 17.4) and TNF-α (OR = 0.19, 95% CI 0.31, 0.7), as well as the pressure diastolic (OR = 2.1, 95% CI 1.41, 12.1). Therefore, the concentration of fibrinogen, systolic and diastolic pressure of cases during the exposure to dust storm was significant. Also, the maximum correlation was estimated between the fibrinogen and As (R = − 0.7) and Mg (R = − 0.64). Whereas there was a negative relationship between TNF-α concentration and Pb during the normal day (R = − 0.85). On the other hand, the maximum correlation between heavy metals on a normal day and a dust storm was related to Cd and Zn (R = 0.96) and Cu and Mg (R = 0.88), respectively. There is a significant relationship between the concentrations of heavy metals during exposure to a dust storm which can interfere with the level of cardiovascular factors. Therefore, it is essential to improve the building architecture and ventilation system to prevent the emission of dust into indoor air and reduce human exposure.

## Supplementary Information

Supplementary Table S1.

## Data Availability

The data supporting the results of this study are available within the article.
