# Supplementary material for: Short-term exposure to some heavy metals carried with PM10 and cardiovascular system biomarkers during dust storm
Source: Sci Rep. 2023 Apr 15;13:6146. doi: 10.1038/s41598-023-31978-x (PMC10105359; doi:10.1038/s41598-023-31978-x)
Supplement: Supplementary file 1 — Supplementary Table S1. [file 41598_2023_31978_MOESM1_ESM.docx]

Table S1. The properties of investigated cases

| **Age (year)** | **20-30** | | | | | | **30-40** | | | | | | | **40-50** | | | | | **50-60** | | | | |
| --- | --- | --- | --- | --- | --- | --- | --- | --- | --- | --- | --- | --- | --- | --- | --- | --- | --- | --- | --- | --- | --- | --- | --- |
|  | 29% | | | | | | 27% | | | | | | | 36% | | | | | 8% | | | | |
| **Education** | **High school** | | | | | | **Diploma** | | | | | | | **Bachelor** | | | | | **MSc or phD** | | | | |
|  | 75% | | | | | | 14% | | | | | | | 9% | | | | | 2% | | | | |
| **BMI** | **<18** | | | | | | **18-21** | | | | | | | **21-25** | | | | | **>25** | | | | |
|  | 4% | | | | | | 25% | | | | | | | 36% | | | | | 35% | | | | |
| **Exposure to outdoor (hr)** | **<3** | | | | | | **4** | | | | | | | **5** | | | | | **>6** | | | | |
|  | **Normal day** | | **Storm day** | | | | **Normal day** | | **Storm day** | | | | | **Normal day** | **Storm day** | | | | **Normal day** | **Storm day** | | | |
|  | 66% | | 75% | | | | 16% | | 9% | | | | | 5% | 5% | | | | 13% | 11% | | | |
| **Exposure to occupational pollution (hr)** | **0** | | | | | | **<3** | | | | | | | **3-4 hr** | | | | | **4-5** | | | | |
|  | 100% | | | | | | 0 | | | | | | | 0 | | | | | 0 | | | | |
| **Sex** | **Men** | | | | | | | | | | | | | **Women** | | | | | | | | | |
|  | 93% | | | | | | | | | | | | | 7% | | | | | | | | | |
| **Use of mask** | **Yes** | | | | | | | | | | | | | **No** | | | | | | | | | |
|  | 10% | | | | | | | | | | | | | 90% | | | | | | | | | |
| **Type of mask** | **No** | | | | | | **3 layers** | | | | | | | **Protective for PM** | | | | | **Cartridge respirator** | | | | |
|  | **Normal day** | | | | | **Storm day** | **Normal day** | | | **Storm day** | | | | **Normal day** | | | **Storm day** | | **Normal day** | | **Storm day** | | |
|  | 89% | | | | | 92% | 11% | | | 8% | | | |  | | |  | |  | |  | | |
|  |  | | | | |  |  | | |  | | | |  | | |  | |  | |  | | |
| **Exposure to second smoking (hr)** | **0** | | | | | | **<3** | | | | | | | **3-4** | | | | | **4-5** | | | | |
|  | Normal day | | | **Storm day** | | | **Normal day** | | | | **Storm day** | | | **Normal day** | | | **Storm day** | | **Normal day** | | | **Storm day** | |
|  | 2% | | | 0 | | | 68% | | | | 75% | | | 30% | | | 25% | |  | | |  | |
| **Use of drugs** | **Yes** | | | | | | | | | | | | | **No** | | | | | | | | | |
|  | **Normal day** | | | | | | **Storm day** | | | | | | | **Normal day** | | | | | **Storm day** | | | | |
|  | 18% | | | | | | 4% | | | | | | | 82% | | | | | 96% | | | | |
| **Dose of drugs (dose)** | **0** | | | | | | **1** | | | | | | | **2** | | | | | **3** | | | | |
|  | **Normal day** | | | | **Storm day** | | **Normal day** | | | | | | **Storm day** | **Normal day** | | | | **Storm day** | **Normal day** | | | **Storm day** | |
|  | 83% | | | | 94% | | 13% | | | | | | 3% | 4% | | | | 0 | 0 | | | 3% | |
| **Use of fast food** | **0** | | | | | | **1** | | | | | | | **2** | | | | | **3** | | | | |
|  | **Normal day** | | | | **Storm day** | | **Normal day** | | | | | **Storm day** | | **Normal day** | | | | **Storm day** | **Normal day** | | **Storm day** | | |
|  | 0 | | | | 0 | | 56% | | | | | 93% | | 44% | | | | 7% | 0 | | 0 | | |
| **Distance from traffic (m)** | **<500** | | | | | | **500-1000** | | | | | | | **>1000** | | | | |  | | | | |
|  | 2% | | | | | | 11% | | | | | | | 87% | | | | |  | | | | |
| **Type of traffic** | **Light** | | | | | | **Medium** | | | | | | | **Heavy** | | | | |  | | | | |
|  | 84% | | | | | | 14% | | | | | | | 2% | | | | |  | | | | |
| **Use of vegetables and fruits** | **1** | | | | | | **2** | | | | | | | **3** | | | | |  | | | | |
|  |  |  | | | | |  | | | | | | |  | | | | |  | | | | |
|  | **Normal day** | **Storm day** | | | | | **Normal day** | **Storm day** | | | | | | **Normal day** | | **Storm day** | | | **Normal day** | | | | **Storm day** |
|  | 25% | 61% | | | | | 43% | 29% | | | | | | 27% | | 9% | | |  | | | |  |
| **Type of water** | **Municipal network** | | | | | | | | | | | | | **Potable water** | | | | | | | | | |
|  | 0 | | | | | | | | | | | | | 100% | | | | | | | | | |
